# Understanding Catalyst ‘Volcano’ Dependence Through Fermi-Level Controlled Kinetics Using Electronic Theory

**DOI:** 10.3390/e26121029

**Published:** 2024-11-28

**Authors:** Nigora Turaeva, Gregory Yablonsky, Rebecca Fushimi

**Affiliations:** 1 Department of Natural Sciences and Mathematics, Webster University, Saint Louis, MO 63119, USA; nigoraturaeva82@webster.edu; 2 Department of Energy, Environmental and Chemical Engineering, McKelvey School of Engineering, Washington University in Saint Louis, Saint Louis, MO 63130, USA; 3 Catalysis and Transient Kinetics Group, Idaho National Laboratory, Idaho Falls, ID 83415, USA; rebecca.fushimi@inl.gov

**Keywords:** catalysis, kinetics, Fermi level, volcano-shape

## Abstract

The ubiquitous two-step Michaelis–Menten and Temkin–Boudart reaction mechanisms are extended to include the influence of the catalyst electronic subsystem in a 5-step mechanism. The resulting kinetic equation provides an alternative explanation for the well-known volcano-shaped dependence found in catalysis. The equilibrium constants of fast electronic steps are highlighted for their influence on adsorption and desorption through the relative concentration of charged versus neutral intermediates. This generalized concept can be widely applied to determine the optimal catalyst, based on the Fermi level of the material, for reactions proceeding via this universal reaction.

## 1. Introduction

The concept of the electronic control of catalytic activity by tailoring the Fermi level was formulated by Th. Wolkenstein more than 70 years ago [1]. According to this theory, a chemisorbed molecule is considered a structural defect of a semiconductor material. Consequently, the localized energy levels of the adsorbate are introduced into the bandgap of the semiconductor. Localization of free electrons or holes on the chemisorbed molecule changes the character of its chemical bond with the surface via this mechanism, see also the detailed discussion in [2,3]. The catalytic activity of a semiconductor catalyst is determined by the Fermi level of the material. More specifically, several characteristics of chemical reactions catalyzed by semiconductors will therefore depend upon the Fermi level of the catalyst: (1) a relative number of chemisorbed forms at electronic equilibrium, hence the reactivity; (2) surface absorptivity for each species and the catalytic activity with respect to a given chemical reaction; (3) selectivity of a catalyst with respect to two (or more) concurrent reactions. The electronic theory of catalysis by semiconductors establishes a correlation between the catalytic activity and electrical conductivity of semiconductors since both are determined by the Fermi level. All external factors that impact the Fermi level characteristics, including catalyst doping, dispersion, illumination, electric field, etc., change the catalytic activity by accelerating or retarding specific reactions and, as such, modify the chemical kinetic properties of the active material.

Wolkenstein’s original theory, developed for semiconductors, cannot be directly applied to metal catalysts since metals have no bandgap and the origin of chemical bonds with adsorbates is different. To overcome this limitation, the d-band model developed by Hammer and Nørskov [4], which explains the formation of chemical bonds between the molecular orbitals of adsorbates and the narrow d-band energy states of transition metals, has recently been applied [2,3]. According to d-band theory, the location of the Fermi level characterizes the degree to which antibonding states are occupied and, hence, the strength of the bond between the adsorbate and the metal surface. Presently, it is topical to develop generalized kinetic consequences of the Fermi level for this electronic concept via a robust and broadly utilized catalytic mechanism, i.e., the Michaelis–Menten and Temkin–Boudart mechanisms.

## 2. The Simplest Mechanisms of Catalytic Systems: Michaelis–Menten and Temkin–Boudart Mechanisms

The Michaelis–Menten and Temkin–Boudart mechanisms are widely utilized in enzyme catalysis and heterogeneous “gas-solid” catalysis, respectively. These mechanisms are minimal and identical from the point of formal kinetics. For these two mechanisms we propose to use one term, the Michaelis–Menten–Temkin–Boudart (MMTB) mechanism.

Every problem has its own history. Traditionally, this mechanism is attributed to Michaelis and Menten in 1913 for enzyme kinetics [5,6] presenting it as (1) E + S = ES; (2) ES = P + S, where S and P are substrate and product, respectively, and E and ES are different forms of enzyme and enzyme-substrate complexes, respectively. In 1925, Briggs and Haldane [7] analyzed this mechanism and its corresponding kinetic equation (see in detail [8]). Later, Temkin formulated this mechanism for heterogeneous catalysis as follows: (1) A + Z = AZ + C; (2) AZ + B = Z + D, where A and B are reactants; C and D are products; Z and AZ are a catalytic reaction site and adsorbed reactant on such site, respectively. Then, Boudart popularized it as a basic mechanism of heterogeneous catalysis and applied it to many catalytic reactions [9].

The MMTB-mechanism is generally conceptualized as two steps: reactant adsorption and product desorption with one common intermediate. In this work, we extend this general mechanism into electronic theory with the goal of explaining the well-known Sabatier principle, manifested as the ‘volcano’ dependence in heterogeneous catalysis [10]. In accordance with Gorban’s analysis [8,11], the first extension of the two-step mechanism was performed more than a hundred years ago by Michaelis and Menten themselves. They proposed the mechanism of type: (1) E + S = ES; (2) ES = PS; (3) PS = P + S. Also, Michaelis and Menten assumed that the compound ES is in partial equilibrium with E and S, whereas PS is in partial equilibrium with P and S. In 1952, Stueckelberg [12] used the same assumptions or the derivation of the Boltzmann equation from Markovian microkinetics. Gorban used the extended mechanism, i.e., the three-step MMTB-mechanism, for solving the paradox of the transition state theory [11]; see [13] as well.

In this work, we extend this mechanism further to reflect three additional important features:

1. Adsorbed intermediates associated with reactants and products are distinct, e.g., AZ vs. PZ;
2. These adsorbed intermediates are transformed into charged species in accordance with Wolkenstein’s theory;
3. In some reactions, one charged compound may be transformed into another charged species.

So, the extended five-step Michaelis–Menten–Temkin–Boudart (MMTB)-mechanism will be as presented in Table 1, which is characterized by the features mentioned above. More specifically, in this mechanism we identify:

- Three specific intermediates, two adsorption–desorption intermediates in a neutral state, AZ^0^, PZ^0^, are related to the reactant adsorption (step 1) and product desorption (step 5), respectively. The third is simply an active site Z.
- Steps of electronic transfer (steps 2 and 4) are based on Fermi-level considerations. Two “adsorption–desorption” intermediates, AZ^0^ and PZ^0^, are transformed into charged intermediates via electronic transfer to AZ^−^ and PZ^−^, respectively.
- The transformation of two charged intermediates takes place, i.e., AZ^−^ is transformed into PZ^−^ in Step 3.

Returning to Wolkenstein’s original theory, we recall that weak chemisorption of molecules takes place without participation of free valences on the surface. Desorption of a species only occurs when they are in a weak chemisorption form. In a strong chemisorption state, free electrons and holes of the catalyst participate in the formation of chemical bonds with molecular orbitals of adsorbed molecules. Electrons of the catalyst are responsible for forming the acceptor bonds with the adsorbed molecules while holes of the surface form donor bonds. The occupancy of the localized energy states of intermediates adsorbed on the surface by electrons or holes follows the Fermi–Dirac statistics, and the fractions of weak and strong chemisorbed intermediates on the surface are determined by the Fermi level of the catalyst. Since these intermediates exhibit different reactivity with respect to a specific reaction, the catalytic activity becomes a function of the Fermi level of the catalyst.

The turnover frequency (TOF), a fundamental concept of heterogeneous catalysis, in the theory of steady-state reaction is determined as follows:

$$TOF=\frac{R}{N_{Z}}$$

where *R* is the reaction rate of the complex catalytic reaction obtained under quasi-steady-state assumptions (molecule/cm^2^/s) and *N_z_* is the total number of active sites per unit area (molecules/cm^2^); thus, the dimension of TOF is *s*^−1^. Consequently, 1/TOF = $N_{z}/R$ with dimension of *s*. For our mechanism in Table 1, under the same quasi-steady-state assumptions using the modified form of the rate of the complex catalytic reaction [14], we find:

(1) $$1/TOF=1/{\tilde{k}}_{1}+(1/k_{2})\left(1+1/{\tilde{K}}_{1}\right)+(1/k_{3})(1+1/K_{2}+1/(K_{2}{\tilde{K}}_{1}))\;+(1/k_{4})\left(1+1/K_{3}+1/(K_{3}K_{2}\right)+1/(K_{3}K_{2}{\tilde{K}}_{1}))\;+(1/k_{5})\left(1+1/K_{4}+1/(K_{4}K_{3}\right)+1/(K_{4}K_{3}K_{2})+1/(K_{4}K_{3}K_{2}{\tilde{K}}_{1}))$$

Here $k_{j}$ and $k_{j-}$ are kinetic coefficients of the *j*-th forward and reverse step, respectively; ${\tilde{k}}_{1}$ is the apparent kinetic coefficient of the 1st step, $K_{j}=k_{j}/k_{j-}$ is the equilibrium constant of the *j*-th step and ${\tilde{K}}_{1}$ is the apparent equilibrium constant of the 1st step. Apparent kinetic coefficients and apparent equilibrium constant may include concentrations of components, reactants or products, as factors. Here ${\tilde{k}}_{1}=C_{A}k_{1}$ and ${\tilde{K}}_{1}={\tilde{k}}_{1}/k_{1-}$. For some purposes, the characterstic 1/TOF appears more advantageous than the traditional characteristic turnover frequency widely advertised by Boudart.

First, 1/TOF reflects the structure of the detailed mechanism in a simple way. In contrast to the chemical reaction rate *R* and TOF = *R*/*N_z_* which are nonlinear characteristics (fractions), the main property of 1/TOF is its additivity. 1/TOF is a linear combination of terms, each of which corresponds to a specific reaction step. Therefore, it is more convenient to study the influence of different steps on the complex reaction rate using the form 1/TOF as in Equation (1). This characteristic, 1/TOF can be described as a ‘chemical resistance’ reflecting the sequence of steps, similar to the equivalent resistance of resistors connected in series in an electronic circuit. Second, every term of Equation (1) typically consists of two factors, one kinetic and one thermodynamic. The kinetic factor is the reciprocal kinetic coefficient (apparent kinetic coefficient) of the *j*-th forward reaction. The thermodynamic factor of a term is a simple function of equilibrium constants (apparent equilibrium constants) of other steps. It depends on the reversibility of the mechanism.

Considering kinetic coefficients of electronic steps 2 and 4 to be very fast, Equation (1) is simplified:

(2) $$1/TOF=1/{\tilde{k}}_{1}+(1/k_{3})(1+1/K_{2}+1/(K_{2}{\tilde{K}}_{1}))\;+(1/k_{5})\left(1+1/K_{4}+1/(K_{4}K_{3}\right)+1/(K_{4}K_{3}K_{2})+1/(K_{4}K_{3}K_{2}{\tilde{K}}_{1}))$$

It can also be presented as a sum of different terms by factoring electronic equilibrium constants:

(3) $$1/TOF=1/{\tilde{k}}_{1}+1/k_{3}+1/k_{5}+1/k_{3}\;[1/K_{2}\;(1+1/{\tilde{K}}_{1})]+1/k_{5}\{1/K_{4}\;[1+1/K_{3}\;(1+1/{\tilde{K}}_{1})]\}\;+1/k_{5}[1/(K_{4}\;K_{3}\;K_{2})](1+1/{\tilde{K}}_{1})$$

This kinetic expression does not include the fast kinetic parameters of electronic transfer; however, it includes the equilibrium constants of electronic steps 2 and 4 which significantly influence the conclusions. Therefore, electronic steps, despite being very fast, remain kinetically significant for describing steady-state/quasi-steady state kinetic behavior. This is an important point since the electronic subsystem, much faster than atomic restructuring, has been previously disregarded. Indeed, forward and reverse electronic steps are fast but their balance in the equilibrium constants *K*_2_ and *K*_4_ is what determines the relative concentrations of charged versus neutral intermediates.

In Equation (3), the terms of $1/{\tilde{k}}_{1}$, $1/k_{3}$, and $1/k_{5}$ do not contain electronic equilibrium constants. The terms of 1/k_3_ [1/K_2_ (1 + 1/${\tilde{K}}_{1}$)] and 1/k_5_[1/K_4_ [1 + 1/K_3_ (1 + 1/${\tilde{K}}_{1})$]] reflect the transfer of electrons between the catalyst and the acceptor intermediates via step 2 and between the donor intermediates and the catalyst via step 4, respectively. Also, the last term 1/k_5_[1/(K_4_ K_3_ K_2_)](1 + 1/${\tilde{K}}_{1})$ in the sum reflects the transfer of electrons between the catalyst and both acceptor and donor intermediates via steps 2 and 4. The equilibrium constants of electronic steps determine the ratios of adsorbed species in weak and strong chemisorbed states and, following Fermi–Dirac statistics, depend on the energy difference between the Fermi level of the catalyst and antibonding molecular orbitals of AZ and PZ compounds, respectively:

$$K_{2}=\frac{k_{2}}{k_{-2}}=\frac{N_{AZ}^{-}}{N_{AZ}^{o}}=e^{-\frac{E_{F}-E_{ab}^{AZ}}{kT}}\;K_{4}=\frac{k_{4}}{k_{-4}}=\frac{N_{PZ}^{o}}{N_{PZ}^{-}}=e^{\frac{E_{F}-E_{ab}^{PZ}}{kT}}$$

where *N_AZ_* and *N_PZ_* represent the number of AZ and PZ surface intermediates in either the strong ($N_{AZ}^{-}$, $N_{PZ}^{-})$ or weak ($N_{AZ}^{o}$, $N_{PZ}^{o})$ chemisorbed state, $E_{F}$ is the Fermi level of the catalyst, $E_{ab}^{AZ}$ and $E_{ab}^{PZ}$ are the energies of antibonding molecular orbitals of AZ and PZ compounds, respectively. We can then rewrite Equation (3) as a function of the catalyst Fermi level with respect to the antibonding molecular orbitals of AZ and PZ compounds:

(4) $$\begin{array}{cc} 1/TOF=1/{\tilde{k}}_{1} & +1/k_{3}\left(1+\left(1+1/{\tilde{K}}_{1}\right)e^{\frac{E_{F}-E_{ab}^{AZ}}{kT}}\right)\\ & +1/k_{5}\left(1+\left(1/K_{3}+\left(1/{\tilde{K}}_{1}\right)\left(1/K_{3}\right)\right)e^{\frac{E_{ab}^{PZ}-E_{ab}^{AZ}}{kT}}+\left(1+1/K_{3}\right)e^{-\frac{E_{F}-E_{ab}^{PZ}}{kT}}\right) \end{array}$$

here $e^{\frac{E_{ab}^{PZ}-E_{ab}^{AZ}}{kT}}$ is the product of two equilibrium constants of electronic steps.

Analyzing Equation (4), it is easy to show that the extremum of 1/TOF is achieved at the optimal Fermi level:

(5) $$E_{F}^{opt}=\frac{1}{2}\left[E_{ab}^{PZ}+E_{ab}^{AZ}\right]+\frac{kT}{2}ln\left[\left(\frac{k_{3}}{k_{5}}\right)\left(\frac{1+1/K_{3})}{1+1/{\tilde{K}}_{1}}\right)\right]$$

In Equation (4) the exponential functions have the opposite sign of the Fermi level, hence a nonmonotonic kinetic dependence is expected. The second derivative of 1/TOF regarding the value of *E_F_* is positive:

$$\frac{\partial^{2}}{\partial E_{F}}\left(\frac{1}{TOF}\right)=\frac{1}{{\left(kT\right)}^{2}}\left[\frac{1}{k_{3}}\left(1+\frac{1}{{\tilde{K}}_{1}}\right)e^{\frac{E_{F}-E_{ab}^{AZ}}{kT}}+\frac{1}{k_{5}}\left(1+\frac{1}{K_{3}}\right)e^{-\frac{E_{F}-E_{ab}^{PZ}}{kT}}\right]>0$$

and therefore, the extremum of 1/TOF is a minimum. Consequently, the extremum of TOF is the maximum. It means that the dependence of TOF on the Fermi level of the catalyst has a volcano shape, which is a subject of great interest in many areas of heterogeneous catalysis.

Within classic catalytic theory, the optimal relationships have been interpreted based on the linear Brønsted−Evans−Polanyi (BEP) equation [15]; see [16] as well. Within our Fermi-level based concept, volcano-shaped dependence is understood using an alternative explanation based on competing electronic transfers between the catalyst and the adsorbed intermediates AZ and PZ. Previously, we have applied these electronic concepts to different catalytic mechanisms, i.e., the 6-step ammonia decomposition mechanism [2] and to both 3- and 4-step mechanisms of CO oxidation [3]. In all cases, the volcano-shaped dependences were demonstrated.

Equation (4) has a clear physicochemical meaning: it provides guidance for selection of the optimal catalyst for a given catalytic reaction. The Fermi level of such a catalyst can be estimated as the arithmetic mean of energies of antibonding molecular orbitals of intermediate compounds. The last term of Equation (5), $\frac{kT}{2}ln\left[\left(\frac{k_{3}}{k_{5}}\right)\left(\frac{1+1/K_{3})}{1+1/{\tilde{K}}_{1}}\right)\right]$, is a function of the temperature and reactant concentration. If parameters $k_{3}\;\mathrm{and}\;k_{5}$, $K_{3}\;\mathrm{and}\;{\tilde{K}}_{1}$ have the same order of magnitude, this term can be neglected. Another goal is related to the search for the optimal temperature regime for the given catalyst/catalytic reaction that reflects the detailed mechanism with estimates of its parameters and Fermi-level considerations. However, the analysis of this problem is a topic for future work.

It is well known that kinetic dependence can be simplified based on the type of catalyst and/or the process conditions for the same catalyst. In Equation (4), we can consider two possible limiting cases: (1) the rate is limited by the adsorption of reactants; (2) the rate is limited by the desorption of products. We assume that $1/{\tilde{k}}_{1}$ and $1/k_{5}$ are negligible, since intermediates in weakly chemisorbed states adsorb and desorb quickly for all transition metals. In the first case, when the rate is limited by the adsorption of reactants, we omit the term involving $e^{-\frac{E_{F}-E_{ab}^{PZ}}{kT}}$ in the summation, as it represents the reciprocal of $K_{4}$ and corresponds to the large fraction of weakly chemisorbed products ($K_{4}=\frac{N_{PZ}^{o}}{N_{PZ}^{-}}\gg 1$). Then, we have

(6) $$\frac{1}{TOF}=1/k_{3}+1/k_{3}\left(\left(1+\frac{1}{{\tilde{K}}_{1}}\right)e^{\frac{E_{F}-E_{ab}^{AZ}}{kT}}\right)$$

As seen in Equation (6), the TOF decreases as the Fermi level increases, which determines the descending side of the volcano curve. In the case of a rate limit by desorption of products, in Equation (4), the term involving $e^{\frac{E_{F}-E_{ab}^{AZ}}{kT}}$ is negligible as it represents the reciprocal of $K_{2}$ and corresponds to the large fraction of strong chemisorbed reactants ($K_{2}=\frac{N_{AZ}^{-}}{N_{AZ}^{o}}\gg 1$). Therefore, we have

(7) $$\frac{1}{TOF}=1/k_{3}+1/k_{5}\left(1+\frac{1}{K_{3}}\right)e^{-\frac{E_{F}-E_{ab}^{PZ}}{kT}}$$

In Equation (7), we retain the term with $1/k_{5}$, because the exponential function associated with 1/$K_{4}$ is quite large. In this limiting case, the TOF increases with the Fermi level, which defines the ascending side of the volcano curve.

It is important to note that the d-band model for transition metals offers a fundamental framework for connecting the catalytic properties of metals to their electronic structure, including factors such as composition, strain, and surface facets. Norskov and coworkers showed that the binding energy of intermediates serves as a reliable indicator of a catalyst’s reactivity [16]. Additionally, they established correlations between the reactivity of a catalyst and its electronic structure, as well as the behavior of adsorbed intermediates. In a study by Norskov and coworkers [17], based on DFT calculations, it was shown that the electronic structure of adsorbed oxygen serves as a key indicator of its reactivity. In the presented model, we obtained an analytical expression that relates the turnover frequency to the electronic properties of both the metal catalyst (Fermi level) and the intermediates (LUMOs of the reactants and products).

## 3. Conclusions

The extension of two traditional catalytic mechanisms, Michaelis–Menten and Temkin–Boudart mechanisms, was presented as a 5-step electronic mechanism which includes steps of a Fermi-assisted charging of surface intermediates. Considering the equilibrium constants of steps of electronic transfer, a detailed kinetic equation was obtained and then simplified within this generalized concept. Based on this equation, an expression describing the volcano-shaped dependence of catalytic activity was presented. The optimal energy of the Fermi-level was presented for selecting the ideal catalyst working within this ubiquitous reaction mechanism.

## Figures and Tables

**Table 1:** Electronic Michaelis–Menten–Temkin–Boudart (MMTB) mechanism.

$Z+A\begin{array}{c} k_{1}\\ ⇄\\ k_{-1} \end{array}AZ^{0}$	Step (1)
$AZ^{0}\begin{array}{c} k_{2}\\ ⇄\\ k_{-2} \end{array}AZ^{-}$	Step (2)
$AZ^{-}\begin{array}{c} k_{3}\\ ⇄\\ k_{-3} \end{array}PZ^{-}$	Step (3)
$PZ^{-}\begin{array}{c} k_{4}\\ ⇄\\ k_{-4} \end{array}PZ^{0}$	Step (4)
$PZ^{0}\overset{k_{5}}{\to }Z+P$	Step (5)

## Data Availability

The original contributions presented in this study are included in the article. Further inquiries can be directed to the corresponding author.
